# Insights from mouse models into human retinoblastoma

**DOI:** 10.1186/1747-1028-3-9

**Published:** 2008-05-19

**Authors:** David MacPherson

**Affiliations:** 1 Department of Embryology, Carnegie Institution, Baltimore, MD, USA

## Abstract

Novel murine models of retinoblastoma based on *Rb *gene deletion in concert with inactivation of *Rb *family members have recently been developed. These new *Rb *knockout models of retinoblastoma provide excellent tools for pre-clinical studies and for the exploration of the genetics of tumorigenesis driven by *RB *inactivation. This review focuses on the developmental consequences of *Rb *deletion in the retina and the genetic interactions between *Rb *and the two other members of the pocket protein family, p107 (*Rbl1*) and p130 (*Rbl2*). There is increasing appreciation that homozygous *RB *mutations are insufficient for human retinoblastoma. Identifying and understanding secondary gene alterations that cooperate with *RB *inactivation in tumorigenesis may be facilitated by mouse models. Recent investigation of the *p53 *pathway in retinoblastoma, and evidence of spatial topology to early murine retinoblastoma are also discussed in this review.

## Introduction

Retinoblastoma is the most common pediatric primary intraocular tumor. Study of retinoblastoma, a malignant cancer that occurs unilateral or a bilateral form, led to the "two-hit hypothesis" now applicable to other inherited cancer syndromes [1]. In bilateral retinoblastoma, an inherited or *de novo *germline mutation in one *RB *allele is typically observed, with such mutations identified in approximately 90% of patients, and the second *RB *allele undergoes somatic mutation [2,3]. In sporadic, unilateral retinoblastoma, somatic mutations occur in both *RB *alleles. The *RB *gene was the first tumor suppressor gene cloned [4], and *RB *is now known to be mutated not only in retinoblastoma but in many cancers, including osteosarcoma, soft tissue sarcoma, small cell lung, breast, brain, prostate, leukemia, and other sporadic cancers [5-9]. Patients with germline *RB *mutation are at increased risk for many of these tumor types and for melanoma [10-12]. They are also at risk for "trilateral retinoblastoma", involving a pediatric midbrain tumor (pinealoblastoma or suprasellar primitive neuroectodermal tumor) in addition to retinoblastoma [13,14]. Even though *RB *is mutated widely in various human tumor types, the retina is exquisitely sensitive to cancer upon *RB *loss and the reason for this is tissue sensitivity is still poorly understood. Our current knowledge of *RB *function places this gene in pathways central to growth control not only in the retina, but in all tissues. *RB *is a prototypic tumor suppressor gene that encodes pRB, a nuclear phosphoprotein implicated in cell cycle control, differentiation, apoptosis, and many other biological processes. Despite intensive study of this tumor suppressor gene since its cloning, the critical functions of pRB important for tumor suppression are still being unraveled.

### Biological functions of the pRB family

pRB is one member of the "pocket proteins", a family that also includes p107 and p130. These proteins are named owing to the presence of a conserved pocket domain, a region that binds E2F transcription factors and various critical protein interactors, many containing an LxCxE motif. The three pocket proteins exhibit similarities, particularly in the A and B pocket, but there are also clear differences. p107 and p130 have much closer similarity to each other than either to pRB. The pocket proteins differ in their expression patterns, with p107 highly expressed in cycling cells, and p130 expressed at higher levels in cells that have exited the cell cycle [15]. Critical to the function of the pocket proteins are the E2F transcription factors [16]. Pocket proteins can inhibit E2F transactivation activity directly by interfering with the E2F transactivation domain. Also, pocket proteins form complexes with histone deacetylases, histone methyltransferases, histone demethylases, and other chromatin modulators, which act to confer a repressive chromatin state around E2F target genes. There are now 8 known E2Fs, including E2F1 through 8. E2Fs 1–5 act in concert with a DP protein partner to bind the pRB family; pRB binds the so-called activating E2Fs, E2F1, 2, 3a, which have a nuclear localization signal and are strong transcriptional activators. pRB also binds E2F4. p107 and p130 bind E2F4 and 5, often referred to as repressive E2Fs, although p107 and p130 can also form complexes with activating E2Fs under some conditions [17]. E2Fs 6–8 do not function through pocket protein binding. Pocket protein/E2F complexes are dynamic and change upon progression through the cell cycle [18]. The extent to which specific E2Fs mediate pocket protein function and whether E2Fs are the most critical effectors are important questions. The three pocket proteins differ in many binding partners in addition to E2Fs. For example, p130 but not pRB, interacts with members of the DREAM complex, which binds to many promoters and acts to repress cell cycle genes in quiescent cells [19-21]. Notably, this complex includes homologs of *C elegans *SynMuvB genes (important for vulval development) and *Drosophila melanogaster *dREAM (drosophila RBF, E2F and Myb-interacting) complex members. There is also evidence that pRB may bind pro- differentiation factors, such as CBFA1, MITF, C/EPBs and MyoD to promote cell type specific differentiation [22-25]. Data implicating pRB in differentiation control suggest that pRB functions as a positively activating transcriptional co-activator, which is very different from the well-characterized repressive effects of pRB on E2F activity in cell cycle control. The specific effectors for *RB *family members that contribute to retinoblastoma are not well understood. Here, we focus on insights that study of the murine *Rb *family function in the retina *in vivo *have for understanding human retinoblastoma.

### Retinal Development

**Figure 1:**
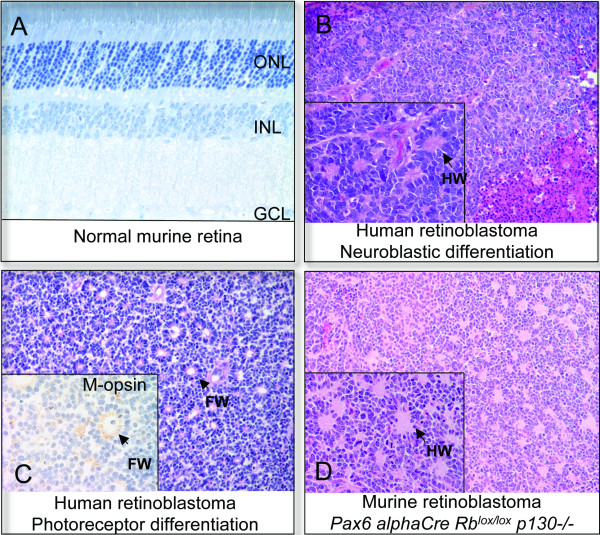
**Normal retina and retinoblastoma (A) **Thin section of normal murine retina with outer nuclear (ONL), inner nuclear layer (INL) and ganglion cell layer (GCL) shown. **(B) **Hematoxylin and Eosin (H+E) stain of human retinoblastoma with neuroblastic differentiation and Homer-Wright (HW) rosettes. **(C) **H+E staining of human retinoblastoma exhibiting Flexner-Wintersteiner (FW) rosettes. Inset: M-opsin immunostaining showing evidence of cone differentiation. **(D) **H+E of primary murine retinoblastoma lacking *Rb *and *p130 *with Homer-Wright rosettes indicated.

The neural retina is composed of seven classes of cell types derived from a common population of progenitors, with cell bodies organized into three nuclear layers. The outer nuclear layer contains cell bodies for rod and cone photoreceptors; the inner nuclear layer contains bipolar, amacrine, horizontal and Müller glia cell bodies, and the ganglion cell layer is made up of ganglion and amacrine cells (Fig 1A). Development of the retina occurs in embryonic and postnatal stages in mice; in humans, retinogenesis is thought to cease near birth [26,27]. The retina derives from anterior tube neuroepithelium that undergoes a lateral bulge after neural tube closure, forming the optic vesicle (reviewed in [28]). Invagination of the central part of the optic vesicle leads to formation of a bilayered optic cup, with an inner layer that forms neural retina, and an outer layer that forms the retinal pigment epithelium. Progenitor cells undergo mitosis along the outer edge of the retina in the retinal ventricular zone. Interkinetic migration occurs such that nuclei migrate away from this zone to undergo G1, S and G2 cycle stages and return for the next mitosis [27]. Lineage tracing studies using retroviral vectors have revealed that mature retinal cell types derive from multipotent progenitors. Potency differs as development proceeds, such that embryonic mitotic clones can contain almost all possible cell types, while postnatal clones are more restricted [29,30]. However, even at late postnatal stages in rodents, 2-cell clones can contain both a Müller glia cell and a rod photoreceptor illustrating multipotency even up until the last cell division [30]. Following cell cycle exit, cells differentiate, migrate to the correct layer and establish appropriate synaptic connections. The developing retina is a simple and very well characterized region of the central nervous system with a defined order of cell type generation, markers for each cell type, and spatial localization of cells at different stages of the cell cycle. Also, the retina is accessible to manipulation *in vivo *using plasmid electroporation, or, if stable expression over many cell divisions is needed, viral vectors. These properties make the retina an ideal system to study not only development, but also cancer initiation. For childhood cancers these processes are clearly intertwined.

### Human retinoblastoma

Retinoblastoma is a pediatric cancer that is typically detected in the first few years of life but can arise during embryonic retinal development [31]. The identity of the specific cell from which retinoblastoma arises is unknown. It has been proposed that retinoblastoma arises from a primitive neuroectodermal cell with multipotent potential [32]. Histologically, retinoblastomas exhibit neuronal differentiation and exhibit focal regions that contain rosettes. Homer-Wright rosettes, indicative of neuroblastic differentiation are present in retinoblastoma and various neuroectodermal tumors (Fig 1b). Flexner-Wintersteiner rosettes exhibit ultrastructural evidence of photoreceptor differentiation [33,34]. Primitive outer segments in such rosettes stain positively for markers of photoreceptor antigens such as interphotoreceptor binding protein (IRBP) and opsins (See Fig. 1c). The rod photoreceptor rhodopsin was found in Flexner-Wintersteiner rosettes in most tumors examined [35,36] and, in a separate study, both rod and cone markers were expressed in many retinoblastomas, with increased staining of cone markers over rod markers [37]. The stronger cone character of retinoblastoma is also supported by studies of a number of cell lines, which revealed mRNA for cone but not rod cGMP phosophdiesterase, and cone transducins [38,39]. Together, these observations have led to a view that retinoblastoma is derived from a developing photoreceptor or a cell with the potential to adopt a photoreceptor cell fate. The question of whether the retinoblastoma-originating cell is a post-mitotic cell specified to become a photoreceptor (i.e. a transition cell), or a restricted progenitor, or a broadly multipotent cell capable of forming many cell types must be resolved.

Lineage tracing studies in the normal rodent retina do not support the existence of specific progenitors that generate only rod and cone photoreceptors [30]. Instead, such studies have revealed that normal retinal progenitor cells are multipotent; clones containing cones, an early born cell type, almost always included different cell types as well [29]. If retinoblastoma is derived from a progenitor cell capable of forming cone photoreceptors, than such a cell would be expected to have broad potency. While much of the focus of retinoblastoma cell of origin studies has been on photoreceptors, inner cell layer markers have also been detected in retinoblastoma [40]. Also, early retinoblastomas sometimes appear to emerge from the inner nuclear layer and not the photoreceptor layer implicating a possible inner layer cell-of-origin [41]. There are many reports describing Müller cells in retinoblastoma [42,43], but it has been debated whether such cells are neoplastic, as opposed to reactive non-tumor cells [44]. Findings that retinoblastoma cells in culture can be induced to express glial markers and exhibit the potential to differentiate into neuron or glial cells support the idea that retinoblastomas arise from multipotent progenitor cells [32]; however, direct evidence of neoplasia in Müller cells from retinoblastoma cells that have not undergone long term culture is needed. Observations that normal progenitor cells in rodents can generate clones containing rods, cones, Müller glia and other cells [29] support the idea that cell type heterogeneity in retinoblastoma may be due to transformation of a progenitor that is normally multipotent, but dedifferentiation or trans-differentiation of a cell-type specified cell-of-origin cannot be ruled out. More recently, stem cell markers have been found expressed in human retinoblastoma cell lines, such as Oct3/4 and Nestin [45,46]. Overall, the human data suggest tumor origin in a multipotent cell but the extent to which these cells are multipotent requires further examination.

There are limitations to using human tumor samples to identify the tumor cell of origin. In the establishment and long term passaging of cell lines, genetic alterations may occur that alter the cell's properties. Also, accessing early retinoblastomas is rarely possible, limiting analysis to late stage tumors that may have quite different characteristics compared to the earliest lesions. Use of model systems can help in assessing the early events in tumorigenesis.

### Transgenic retinoblastoma models

A number of models of retinoblastoma have been developed that have made use of transgenic expression of specific viral oncoproteins. The most widely used such model, the *LH-beta T-Ag *model expresses the SV40 early region under control of the Luteinizing hormone beta promotor [47]. The development of retinoblastomas in one *LH-beta T-Ag *line was in part a consequence of the genomic site of integration directing transgene expression to cells in the retina, a tissue in which luteinizing hormone beta is not normally expressed. Both Large-T and small-t oncoproteins are encoded by the SV40 early region and may contribute to tumorigenesis. Large-T Ag binds the pRB family, p53, and other proteins, such as CBP/p300. Small-t Ag also contributes to transformation in cell culture, in part through binding the phosphatase pp2A [48]. In the *LH-beta T-Ag *model, retinoblastoma with neuronal characteristics and histological similarities to human retinoblastomas emerged. Transgenic mice expressing T-Ag/t-Ag from the *IRBP *promoter have also been generated and develop retinoblastoma [49-51]. IRBP is expressed not only in photoreceptor cells but in some progenitor cells [52], raising the possibility of a progenitor or newly post-mitotic cell of origin. Also, Phenylethanolamine N-methyltransferase-driven T-Ag (*PNMT-T-Ag*) transgenic mice develop retinal tumors that arise at the retina periphery. This spatial localization of retinal tumors in mice occurred despite T-Ag/t-Ag being expressed in amacrine and horizontal cells in both central and peripheral retina [53,54]. The authors suggest that the tumors arise from amacrine or horizontal cells [54] but it is not clear why the tumors were peripheral as these cell types are present throughout the retina.

Papillomavirus E7 protein binds to the pocket protein family, and other effectors, but does not inactivate p53. *IRBP-E7 *mice underwent photoreceptor degeneration, while *IRBP-E7*; *p53-/- *animals were prone to tumors of the retina that arise in the photoreceptor layer [55]. While initially, it was proposed that *p53 *loss suppressed E7-dependent apoptosis, leading to retinoblastoma, *p53 *loss may contribute in other ways, as these tumors are now recognized to emerge in the midst of high levels of apoptosis and photoreceptor degeneration even upon *p53 *deficiency [56]. This distinction is important, as in other mouse models, there is good evidence that functions of *p53 *independent of apoptosis are critical for tumor suppressor ability [57]. This is also relevant to a renewed interest in the role of *p53 *in retinoblastoma discussed further on in this review. Whether the tumors in the *IRBP-E7;p53-/-, LH-beta T-Ag *and *PNMT-Tag *models exhibit a similar cell of origin or if these models represent distinct tumors of different cell types remains to be determined.

Use of transgenic retinoblastoma models has been valuable for the testing of preclinical therapeutics; however, the development of models that target *Rb *but not other effectors of viral oncoproteins has been a clear goal for improvement of mouse retinoblastoma models. The main disadvantage of transgenic models expressing viral oncoproteins concerns the pleiotropic effects of the oncoproteins, which impact upon multiple signaling pathways. These oncoproteins may also have important targets not yet identified. Furthermore, tumors in which p53 is functionally ablated through T-Ag expression are not ideal for testing therapies, whose effectiveness may depend on the p53 pathway being intact [58,59].

### "*Rb *knockout" models of retinoblastoma

The generation of mouse models that, like human retinoblastoma, exhibit *Rb *deletion took a number of years. Three groups inactivated *Rb *in the mouse germline, resulting in embryonic lethality in *Rb-/- *animals and the development of pituitary and thyroid tumors in *Rb+/- *animals [60-62]. Retinoblastomas never occurred in the murine *Rb *heterozygotes, in stark contrast to the high retinoblastoma incidence in humans with germline *RB *mutation. The Jacks and the Berns groups both generated chimeric animals in which *Rb-/- *cells contributed to the retina, but retinoblastomas still did not form [63,64]. With the generation of chimeric animals lacking *Rb *and *p107 *came the realization that mice were capable of developing retinoblastoma dependent on *Rb *deletion [65]. However, the study of large numbers of retinoblastoma-bearing animals was not practical using this chimera model. Indeed, the low recovery of viable chimeras suggested significant developmental lethality upon high contribution of *Rb-/-; p107-/- *cells to the developing embryo.

Nevertheless, the advent of *Cre-lox *technology and *Cre*-expressing transgenic animals with expression in the developing retina paved the way for the generation of breedable *Rb*-knockout models of retinoblastoma. The first attempt, based upon the previous descriptions of photoreceptor characteristics in retinoblastoma used *Cre *regulated by the *IRBP *promoter together with *Rb*^*Lox *^alleles to drive *Rb *deletion in the photoreceptor compartment and in other tissues [66]. These mice did not develop retinoblastoma, even when combined with both *p107 *and *p53 *deficiency, indicating that the tumor-initiating cells in *Rb/p107 DKO *chimeras were not being targeted when these genes were deleted in developing photoreceptors. Breedable *Rb*-knockout models of retinoblastoma were independently generated using *Cre *transgenics with expression driven by *Nestin*, *Chx10 *and *Pax-6 *promoter in progenitor and other cells [67-70]. New retinoblastoma models confirmed the tumor suppressor function of *p107 *first revealed in chimera studies. Mouse models have now revealed that inactivation of the third pocket protein, *p130*, also cooperates very strongly with *Rb *deletion to promote retinoblastomas in both *Rb *knockout and chimeric mice [68,69,71]. A summary of the different *Rb*-knockout and chimeric retinoblastoma models is provided in Table 1. The breedable *Rb *knockout models of retinoblastoma now provide excellent tools to hone in on the cell of origin and interrogate the molecular genetics of retinoblastoma progression.

**Table 1:** *Rb-/- *mouse models of retinoblastoma

**Model**	**Frequency**	**Tumor Characteristics**	**References**
***Rb-/-p107-/- *chimeras**	5/7 mice (1/5 bilateral)	Positive staining for amacrine markers, some Müller cells present	[65]
***Rb-/-p130-/- *chimeras**	5/11 mice (2/5 bilateral)	Positive staining for amacrine markers, some Müller cells present Average 3 months to tumor detection	[71]
***α-Cre; Rb^*lox*/*lox*^; p107-/-***	> 60%	Positive staining for amacrine markers, some Müller cells present Average 280 days to externally visible retinoblastoma	[67, 68]
***α-Cre; Rb^*lox*/*lox*^; p130-/-***	29/29 mice (bilateral)	Positive staining for amacrine and horizontal markers, some Müller cells present Average 128 days to externally visible retinoblastoma Lymph node and brain metastasis	[68]
***NesCre1; Rb^*lox*/*lox*^; p130-/-***	5/5 mice (4/5 bilateral)	Positive staining for amacrine markers, some Müller cells present	[69]
***Chx10Cre; Rb^*lox*/*lox*^; p107-/-***	~60%	Positive staining for amacrine, horizontal and progenitor markers	[89]
***Chx10Cre; Rb^*lox*/*lox*^; p107-/-; p53-/-***	100% (bilateral)	Positive staining for amacrine, horizontal and progenitor markers Increase in less differentiated cells vs. *Chx10Cre; Rblox/lox* *p107-/- *tumors Average 100 days before mice were moribund	[70, 89, 129]
***Chx10Cre; Rb^*lox*/*lox*^; p130-/-;p107+/-***	45/45 mice (bilateral)	Positive staining for horizontal cell markers in primary tumors Metastastes did not stain for horizontal cell markers Tumors arose at 8-14 weeks	[95]

### *Rb *in murine retinal development

Retinoblastoma is a clear example of a tumor with developmental origins and, as such, it's understanding requires knowledge of the normal developmental role of *Rb *in the retina. Homozygous germline *Rb *deletion in mice leads to embryonic lethality in midgestation, caused by placental deficiency [60-62,72]. *Rb *has been inactivated in the retina using a number of different systems that bypass the lethality associated with systemic *Rb *deletion. *Rb-/-;+/+ *chimeric animals and various *Cre*-expressing transgenics, including *Chx10-cre*, *Pax6 α-enhancer Cre *(*α-Cre*) and *NesCre1*, in concert with an *Rb lox/lox *allele exhibit *Rb *deletion in the retina [67,69,73]. These models inactivate *Rb *at different times and in different cells. *Chx10-Cre *is expressed in a mosaic pattern in progenitor cells, and in adults is expressed in most bipolar cells and in some Müller cells [73]. *NesCre1 *is active in the optic vesicle by E9.5 and exhibits different expression upon paternal vs. maternal inheritance of the transgene [69]. Complete recombination in the retina occurs upon paternal *Cre *transmission and is accompanied by lethality at birth in *Rb*^*lox*/*lox *^animals. A mosaic pattern of *Cre *expression is observed with maternal *NesCre1 *inheritance allowing for the survival of some animals on an *Rb*^*lox*/*lox *^background. *α-Cre *is expressed by E10.5 in distal progenitor cells, with a dorsal gap in expression [74]. In addition to the widespread expression in peripheral progenitors, certain cells in the inner nuclear layer and ganglion cell layer express *α-Cre *at late stages both in central and peripheral retina. A synthesis of the data from many groups using different systems has revealed common phenotypes that have implicated *Rb *in control of proliferation, apoptosis and differentiation in the developing retina (Table 2).

**Table 2:** Developmental phenotypes upon *Rb *deletion in the retina

**Genotype**	**Major Developmental Phenotypes**	**References**
***Rb-/- *chimera**	Apoptosis and ectopic mitosis at E16.5–E18.5 in inner layer Loss of most *Rb-/- *cells from all retinal layers in adults	[63]
***Rb-/-p107-/- *chimera**	Apoptosis and retinal dysplasia at E17.5 Photoreceptor death by PND15	[65]
***Rb-/-p130-/- *chimera**	Inner layer proliferation at PND14	[71]
***NesCre1; Rb*^*lox*/*lox*^**	Inner cell layer apoptosis and ectopic BrdU at E18.5 Photoreceptor degeneration in adult mosaic animals	[69]
***NesCre1; Rb^*lox*/*lox*^; p53-/-***	Inner cell cell layer apoptosis and ectopic BrdU at E18.5 Photoreceptor layer degeneration in adult mosaic animals	[69]
***Chx10Cre; Rb*^*lox*/*lox*^**	Defective rod differentiation and rod cell death Ectopic cells in outer plexiform layer	[73, 77, 78]
***Chx10Cre; Rb^*lox*/*lox*^; p107-/-***	Increased proliferation of cells expressing progenitor and amacrine markers at PND30	[84]
***αCre; Rb^*lox*/*lox*^***	Increased apoptosis and proliferation in embryonic inner retina Extension of normal proliferative period of retinal development Ectopic proliferation of transition cells expressing markers of all 7 cell types Death of most bipolar cells, ganglion cells and a subset of rods Defective starburst amacrine cell differentiation	[67-69, 79]
***αCre; Rb^*lox*/*lox*^; p107-/-***	Increased apoptosis and proliferation over *Rb *mutants Death of all peripheral cells except amacrine, horizontal and Müller cells	[67]
***αCre; Rb^*lox*/*lox*^; p130-/-***	Increased proliferation and apoptosis over *Rb *mutants Severe degeneration from PND21 to PND31 Hyperplasia at extreme periphery in all mice by PND31	[68]
***αCre; Rb^*lox*/*lox*^; E2f1-/-***	Rescue of inappropriate proliferation and apoptosis in bipolar, ganglion cells and rods Rescue of electroretinogram response Defects in starburst amacrine cell differentiation remain	[79]
***αCre; Rb^*lox*/*lox*^; E2f2-/-***	Phenotype similar to *Pax6 alphaCre Rblox/lox *retinas	[79]
***αCre Rb^*lox*/*lox*^; E2f3-/- (or E2f3a-/-)***	No rescue of cell death or ectopic cell division Rescue of stabrurst amacrine cell differentiation	[79]

In embryonic stages of retinal development, the first indication that there were cell cycle defects upon *Rb *deletion came from *Rb-/-;Rb+/+*chimeric animals in which mitotic figures and high levels of cell death were found in the inner retina from E16.5 to E18.5 [63]. Ectopic proliferation in the retinal ganglion cell layer has been observed as early as E13.5 in germline *Rb *mutants [75]. Ectopic proliferation coupled to apoptosis in the inner embryonic *Rb-/- *retina was also observed in *α-Cre *and *Nes-Cre1 *mice [67,69]. Normal retinal mitoses are restricted to the outer edge of the retina and never occur in the differentiating inner region suggesting that differentiating *Rb-/- *cells exhibit defects in exiting the cell cycle. The most pronounced effects of *Rb *deletion are seen in late stages of retinal development, as *Rb *loss causes the proliferative period of retinogenesis to be extended [67-69]. Notably, the inappropriately proliferating *Rb-/- *cells at early and late developmental stages express markers associated with differentiation of each of the seven classes of retinal cell types, leading to the idea that *Rb *deletion primarily causes normally post-mitotic, cell-type specified cells (termed transition cells, or precursor cells) to proliferate inappropriately [67]. However, differentiation markers may also be expressed in cells that have not yet committed to a certain cell fate, and it is possible that both progenitor cells and transition cells are affected by *Rb *loss [76]. Importantly, cells in the *Rb*-deficient retina ultimately exited the cell cycle or died such that by PND21, *Rb-/- *cells no longer proliferate, and retinoblastoma never develops [67,68]. Interestingly, the high level of apoptosis that occurs upon *Rb *deletion differentially affects specific cell types. Bipolar cells, ganglion cells as well as the majority of rod photoreceptors are lost in *α-Cre Rb*^*lox*/*lox *^mice. Photoreceptor apoptosis is specific to rods, as cells expressing cone markers were present in normal numbers, revealing specificity to rod cell death [67]. The cell death data are consistent with results from *Rb -/-; +/+ *chimeras, in which there was a low, but not null, contribution of *Rb-/- *cells to the adult retina [63].

Finally, *Rb *loss has been associated with differentiation defects. Rod photoreceptors in *Chx10Cre Rb*^*lox*/*lox*^mice have been reported to exhibit an altered pattern of heterochromatin suggestive of rod maturation defects [73,77,78]. It has been argued that differentiation defects represent direct effects of *Rb *loss, but it is difficult to distinguish between downstream effects of *Rb *loss on cell cycle control vs. direct effects on differentiation. Interestingly, defects in rod development were not found when *IRBP-Cre *was used to drive *Rb *deletion in photoreceptor transition cells [66]. One explanation is that rod differentiation requires pRB only at a progenitor stage prior to IRBP expression but not at later stages. It will be important to precisely determine the specific stage in rod development that is affected. Expression of genes associated with rod synaptogenesis were not altered in *Rb-/- *explant cultures or in *Chx10Cre Rb*^*lox*/*lox *^retinas [78], suggesting that the rods are not completely blocked at a progenitor stage. Nevertheless, some cells express progenitor cell markers in the outer nuclear layer in *Chx10Cre Rb*^*lox*/*lox *^animals [77]; these may be undifferentiated rods, but the possibility that cells other than photoreceptor cells may have inappropriately migrated to the outer nuclear layer complicates their identification. *NesCre1*, *Chx10Cre *and *α-Cre Rb*^*lox*/*lox *^mice all exhibit the presence of ectopic cells in the outer plexiform layer that extend to the photoreceptor layer [69,78], and the origin of these cells has not been determined. Other differentiation defects in *Rb*-mutant retinas have also been noted. *Rb*-deficient starburst amacrine cells were found to lack the expression of markers associated with their maturation [79]. Also, a subset of *Rb*-deficient horizontal cells exhibit enlarged abnormally shaped nuclei, reminiscent of polyploid cells that have undergone endoreduplication in other *Rb*-deficient cell types, such as skeletal muscle and Purkinje cells [64,69]. Defects in *Rb*-deficient horizontal cells involving ectopic processes that extend into the outer nuclear layer have also been described [77].

While there are many similarities between the phenotypes observed following *Rb *deletion using chimera systems and different *Cre*-transgenic lines (Table 2), some differences have also been found. Overall, milder proliferative and apoptotic phenotypes have been reported in studies using *Chx10Cre*-mediated *Rb *deletion versus other systems to inactivate *Rb *in the retina (Table 2). For example, while bipolar cells undergo cell death in *αCre Rb*^*lox*/*lox *^retinas [67,69], this phenotype has not been observed in *Chx10Cre Rb*^*lox*/*lox *^retinas [78]. Differences in genetic background across different strains could contribute to differing phenotypes. It is also possible that differences in the timing of *Cre *expression across various cell types may underlie the differing phenotypes observed. Endogenous retinal *Chx10*, like *Chx10Cre*, is expressed not only in progenitor cells, but, later in retinal development, in mature inner nuclear cells including bipolar cells [73,80]. If heterogeneity in the timing of *Rb *deletion across different cells occurs in animals exhibiting a mosaic pattern of Cre expression, different phenotypes may result. Whether *Rb *is required to maintain cell cycle exit, cell survival and/or differentiation characteristics when deleted late in retinal development is not known and will be important to determine. A second possibility is that in regions of extensive *Rb *deletion, non-cell-autonomous effects contribute to the death of rod, bipolar and ganglion cells. To determine if effects of *Rb *deletion are cell autonomous, the ideal experiment involves stably marking the daughters of mitotic cells using replication-deficient retroviral vectors [81]. Lineage tracing experiments using a retrovirus to express Cre recombinase in newborn animals followed by analysis of clone composition have suggested that defects in rod photoreceptors are cell autonomous [73]. In the same study, while 12 of 115 control clones in *Rb*_*lox*/+_ animals contained bipolar cells following Cre expression, 0 of 82 clones in *Rb*_*lox*/- _animals contained bipolar cells (data from Supplemental Table 2 in [73]). A similar lack of bipolar-containing clones was observed upon functional pRB inactivation through expression of E1A. While larger studies are needed, these numbers suggest that apoptosis is also at least partially cell autonomous in *Rb-/- *bipolar cells. Finally, milder phenotypes may simply be more difficult to detect in animals with a low level of mosaic *Rb *deletion. That is, detecting phenotypes associated with the rapid apoptosis of certain cell types may be facilitated when there is widespread deletion as in *NesCre1 Rb*^*lox*/*lox *^retinas, or in the mid-far periphery of *α-Cre Rb*^*lox*/*lox *^retinas.

The phenotypes upon *Rb *loss are quite similar across studies of chimeras, *α-Cre *and *NesCre1 *mice, with milder phenotypes observed upon *Chx10Cre*-mediated *Rb *inactivation. Differences highlight the need to assess the consequences of *Rb *deletion at different times across different cell types, and the need to better understand the extent to which phenotypes are cell autonomous. Some of these aims may be achieved by expressing *Cre *in a temporally and dosage controllable fashion using either viral delivery or tamoxifen-inducible *Cre-ER *transgenics with *Cre-ER *expression driven by cell-type specific promoters.

### Effectors of *Rb *in the retina

The importance of specific *E2f *family members in mediating *Rb *dependent phenotypes is of great debate. Analyses of *E2f *contribution to *Rb*-mutant developmental phenotypes have been performed in the whole animal through compound mutant studies, but the non-cell autonomous consequences of placental dysfunction in *Rb-/- *embryos complicate the interpretation of these results. Compound mutant analysis specifically in the developing retina provides an excellent system to probe these interactions. The importance of *E2f1 *in mediating the effects of *Rb *loss was nicely shown by the Bremner group as compound mutation of *Rb *and *E2f*1 rescued much of the proliferation and apoptosis associated with *Rb *deletion using the *α-Cre Rb*^*lox*/*lox *^system, and even restored response on the electroretinogram, a measure of retinal electrical activity in response to light [79]. Interestingly, these authors showed that *E2f1 *loss did not rescue differentiation defects in *Rb*-deficient starburst amacrine cells. In contrast, compound mutation of *Rb *and *E2F3a *led to a rescue in starburst amacrine cell development suggesting that *Rb/E2F3a *interactions promote the differentiation of these cells. While pro-differentiation effects of pRB are often thought E2f-independent, this is one example of pRB promoting the differentiation of a cell type mediated through E2F.

It will be critical to dissect the pathway between *Rb *loss and apoptosis in the developing retina. The above study showed that apoptosis is *E2f*-dependent, but the *E2f *effectors that contribute to apoptosis remain to be identified. We were interested in whether *p53 *was downstream of *Rb *inactivation in the retina [69]. Compound *NesCre1 Rb*^*lox*/*lox *^*p53-/- *double mutants did not exhibit rescue of apoptosis in the embryonic retinal ganglion cell layer. Also, the loss of photoreceptors was not rescued in *NesCre1 Rb*^*lox*/*lox *^*p53-/- *animals exhibiting deletion of *Rb *in a mosaic pattern. *Rb *loss has been linked to apoptosis as pRB is an anti-apoptotic protein and many *E2F *target genes are apoptosis effectors [82]. Whether or not it is important to suppress apoptosis for retinoblastoma development is a point of contention, but in many human cancers, tumors may not form unless an apoptotic pathway that is activated following *Rb *deletion is abrogated [83]. Elucidation of the effectors of the *p53*-independent apoptosis downstream of *Rb *loss in the retina will be an important goal for broadly understanding the events that synergize with *Rb *loss to promote cancer.

### Functional overlap among the pocket proteins

Data from chimeras and conditional mutants exhibiting inactivation of *Rb *as well as *p107 *or *p130 *loss clearly illustrate tumor suppressive activity of *p107 *or *p130 *in the context of *Rb *deficiency. It is not clear why retinoblastoma manifests itself in mice only if p107 or p130 is inactivated in addition to pRB. p107 expression is upregulated in murine retinas lacking *Rb *but not in human retina explants with knockdown of RB expression, raising the possibility that p107 upregulation prevents retinoblastoma in mice. [84]. However, increased expression of p107 in *Rb-/- *murine retinal cells has been reported only at postnatal stages of retinal development, or in explant cultures differentiated *in vitro *to recapitulate postnatal stages of retinal development and not in embryonic *Rb-/- *retina [69,84]. Importantly, an increase in proliferation occurs in *Rb-/- *retinas at late time points in retinal development [67-69]. As *p107 *is an *E2f *target gene typically expressed in proliferating but not post-mitotic cells, the *p107 *increase has not been uncoupled from the cell cycle phenotypes associated with *Rb *loss at late stages in retinal development. Other *Rb *targets, such as Cyclin E are found strongly upregulated at both embryonic and postnatal stages of retinal develoment in *Rb*-deficient retinas (D.M. unpublished). It is also possible that functional overlap among the pocket proteins without increased p107 or p130 expression is critical for suppressing retinoblastoma in mice. Indeed, p130 is a stronger suppressor of retinoblastoma than p107 despite no alterations in p130 overall levels upon *Rb *loss [68,69]. Factors that regulate pocket protein activity may differ in their levels or activity in mice compared to humans (either in the normal situation, or in response to *Rb *loss) and many upstream regulators of pocket proteins are known to be important for cell cycle control in the retina. *Cyclin D1-/- *retinas are hypoplastic, corresponding to altered pocket protein phosphorylation [85]. High cyclin D1 levels likely promote normal progenitor cell expansion by keeping the pocket proteins inactive. A decrease in the level of murine cyclin D1 occurs in response to *Rb *family inactivation and this decrease may contribute to retinoblastoma suppression in mice through regulation of pocket protein activity [69]. Indeed, there is a good correlation between the level of cyclin D1 and the extent of p107 phosphorylation in retinas lacking *Rb *or both *Rb *and *p130 *compared to controls. *p27-/- *retinas exhibit an extension in the period of retinogenesis, implicating *p27 *in cell cycle exit in a subset of cells [86]. p19*(Ink4d) *loss also extends the normal period of retinal proliferation in the retina [87]. Whether upstream regulators of p107 and p130 activity such as cyclin D1, p27 and p19Ink4d can contribute to retinoblastoma in the context of *p107 *or *p130 *being intact will be important to elucidate. It is likely that complex differences between mice and humans in the levels and activity of multiple regulators of the RB family contributes to the species difference in retinoblastoma susceptibility. Thus, a broader understanding of the regulation of pRB, p107 and p130 in the cell from which retinoblastoma is derived is needed.

### Genetic interactions between *Rb and p107 *in the retina

Many of the developmental phenotypes associated with retinal *Rb *loss are exacerbated upon additional mutation of p107, revealing the functional overlap among these family members [65,67,69]. Embryonic retinal proliferation and apoptosis are increased upon both *Rb *and *p107 *mutation. While rod and some cone photoreceptors survive when *Rb *is deleted in progenitor cells using the *α-Cre *model, almost all are lost upon additional mutation of *p107 *[67]. Nevertheless, many amacrine cells, horizontal cells and Müller cells survived *Rb *and *p107 *deletion and a subset of such animals developed retinoblastoma. Tumor emergence from highly apoptotic retinas suggests that the cell of origin of retinoblastoma in this model exhibits intrinsic resistant to apoptosis. However, death resistance is a relative term; despite the cell of origin surviving *Rb/p107 *loss, emerging retinoblastomas can be highly apoptotic [68] similar to human retinoblastomas. Secondary alterations that confer evasion of apoptosis may indeed contribute to the progression of retinoblastoma, but this does not diminish the importance of the tumor cell of origin surviving when only three cell types survived in *α-Cre Rb/p107 *DKOs [67]. Retinoblastomas from *Rb/p107 *chimeras or *α-Cre Rb*^*lox*/*lox *^*p107-/- *mice exhibited positive immunostaining for multiple amacrine markers. Amacrine cells are a heterogeneous population with one estimate of 29 subtypes [88]. Heterogeneity in the expression of specific amacrine markers within a tumor and between different tumors suggests that the tumors arise from a cell capable of forming multiple amacrine subtypes. Also, horizontal cells may also be present [89] and some cells expressing glial cell markers [65,67,68] have been observed in retinoblastomas lacking *Rb *and *p107*. Together, these data raise the possibility that tumor cell of origin is capable of generating multiple cell types.

Long latency of tumorigenesis and incomplete penetrance suggests that *Rb *and *p107 *inactivation is not sufficient for retinoblastoma. Thus, the *Rb/p107 DKO *models are ideal for testing genes that potentially cooperate with loss of *Rb *in retinoblastoma development, and to probe the nature of the block(s) that must be overcome.

### Genetic interactions between *Rb and p130 *in the retina

Interactions between *Rb *and *p130 *in the retina have revealed roles for p130 in tumor suppression and retinal development in *α-Cre Rb*^*lox*/*lox *^*p130-/-*, *NesCre1 Rb*^*lox*/*lox *^*p130-/- *and in *Rb-/-p130-/- *chimeras [68,69,71]. In contrast to the embryonic disorganization in *NesCre1 Rb/p107 DKO *retinas, histological defects were not found in *NesCre1 Rb/p130 DKO *embryonic retinas [69]. In postnatal stages of retinal development, proliferation defects are exacerbated by p12 in *α-Cre Rb/p130DKOs*, relative to *α-Cre Rb*^*lox*/*lox *^single mutants, revealing cooperation between *Rb *and *p130 *in controlling cell cycle exit in late stages of retinal development. By PND21, *Rb-/- *cells have all exited the cell cycle, but cells in *α-Cre Rb*^*lox*/*lox *^*p130-/- *retinas continue to inappropriately undergo S-phase entry and exhibit the presence of cells with large aberrant nuclei [68]. Proliferative phenotypes are coupled to high levels of cell death, with severe degeneration in the *α-Cre Rb*^*lox*/*lox *^*p130-/- *retina. The extent to which this cell death is cell autonomous has not yet been determined and it is possible that effects of *Rb *and *p130 *loss on certain cell types may lead to the death of other cells through secondary effects. Cell death is more pronounced in these animals than *α-Cre Rb*^*lox*/*lox *^*p107-/- *retinas, although some cells, including horizontal cells, Müller cells and rare amacrine cells survive at PND21. The survival and increase in horizontal cells at this stage is a specific effect of *Rb *and *p130 *loss not observed upon *Rb *and *p107 *deletion.

Chimeric animals lacking *Rb and p130*, *NesCre1 Rb*^*lox*/*lox *^*p130-/- *and *α-Cre Rb*^*lox*/*lox *^*p130-/- *mice all develop retinoblastomas in which marker expression is heterogeneous and includes the expression of amacrine markers [68,69,71]. Regions of horizontal cell differentiation are also found in *α-Cre Rb*^*lox*/*lox *^*p130-/- *and *NesCre1 Rb*^*lox*/*lox *^*p130-/- *retinoblastomas ([68] and D.M. unpublished). *Rb/p130 DKO *retinoblastomas appear similar to *Rb/p107 DKO *retinoblastomas upon histological examination, and both resemble human retinoblastomas with neuroblastic differentiation (See Figure 1). Early lesions resembling retinoblastoma, with Homer-Wright rosettes, were observed by PND21 to PND31 in *α-Cre Rb*^*lox*/*lox *^*p130-/- *animals at the extreme periphery of the retina [68]. Our observation of peripheral early lesions in *α-Cre Rb*^*lox*/*lox *^*p107-/- *animals points to a possible niche for the cell of origin in both models. This may be similar to the localization of tumors from *PNMT-Tag *animals [53,54] and there also indications that early retinoblastomas in the *LH-beta T-Ag *model preferentially locate to the retina preiphery [90,91]. Notably, despite the death of most amacrine cells away from early tumors at PND21, tumorigenesis is actually more prevalent in the *α-Cre Rb/p130 DKO *model than the *α-Cre Rb/p107 DKO *model, suggesting that the tumors may not derive from amacrine cells or that only a specific subset of peripheral amacrine cells are sensitive to tumorigenesis. [68]. Peripheral progenitor cells/stem cells, Müller glia and horizontal cells are all alternative candidates. Complete retinoblastoma penetrance is striking given the high cell death and suggests that the cell of origin of *Rb/p130 DKO *tumors is less sensitive to apoptosis than most cells in the retina.

*Rb/p130 DKO *retinoblastomas proceed to fill the vitreous, and a subset invade the optic nerve and undergo tumor extension into the brain, similar to advanced human retinoblastoma. Tumors also metastasize to cervical lymph nodes. The *α-Cre Rb/p130 DKO *model is a better model for advanced retinoblastoma given the rapid tumor kinetics. The importance of a mouse model of metastatic retinoblastoma is evident from the poor prognosis of humans with retinoblastoma metastasis [92,93]. In developed countries, retinoblastoma is well treated, with about 4% mortality, however, even with the best care, retinoblastoma with CNS involvement exhibits extremely high mortality [94]. The majority of children with retinoblastoma are born in less developed countries, where delayed retinoblastoma diagnosis leads to extraocular disease; in some parts of the world this results in mortality as high as 90% [94]. Better tumor-eradicating treatments are clearly needed. The advanced tumor progression and the rapid, consistent kinetics in the *α-Cre Rb*^*lox*/*lox *^*p130-/- *animals make this an excellent model for preclinical testing of novel therapies for early and advanced retinoblastoma.

### Effects of *p107 *vs. *p130 *loss in concert with *Rb *in the retina

The differences between *Rb/p107 DKO *and *Rb/p130 DKO *models confirm that *p107 *and *p130 *have differing functions. Distinct cell-type specific effects occur upon *Rb *and *p107 *vs. *Rb *and *p130 *inactivation in the developing retina. Upon *Rb *and *p130 *loss, there is some increase in horizontal cells even in the midst of high levels of retinal degeneration [68]. This phenotype is not observed upon *Rb *and *p107 *loss, implicating an important role for *p130 *in horizontal cell biology distinct from that of *p107*. There is also evidence for an increased horizontal cell component in *Rb/p130 DKO *compared to *Rb/p107 DKO *late stage retinoblastomas [68]. The time to retinoblastoma detection and tumor penetrance differs strongly, with 100% of *Rb/p130 DKO *animals developing bilateral retinoblastoma that emerge rapidly, while only a subset of *Rb/p107 DKO *animals developing unilateral retinoblastoma with long latency. The kinetics of tumorigenesis suggests that there may be a difference in the number of secondary alterations that arise. A critical question is whether the tumors that eventually arise in *Rb/p107 DKO *animals retain normal *p130 *function, or if there is selection to evade a p130-induced differentiation block [67,69].

Despite differences in kinetics and cell type composition, abundant data supports a model of a common cell of origin in *Rb/p130 DKO *vs. *Rb/p107 DKO *retinoblastomas. First, there are similar histological characteristics including the presence of Homer-Wright rosettes [68]. *Rb/p107 *DKO and *Rb/p130 *DKO retinoblastomas both exhibit variable staining for different amacrine cell markers in chimeras, *NesCre1 *and *Pax6 Cre *mice and each of these models exhibit the presence of some glial cells in the tumor. In both *Rb/p107 *and *Rb/p130 *DKO models, we have observed late proliferating cells at the extreme retina periphery. Furthermore, the pattern of tumor dissemination is similar in very advanced tumors, with invasion of the optic nerve and brain, and spread to cervical lymph nodes (although such advanced tumors are rare in *Rb/p107 *DKOs). Finally, secondary alteration analysis reveals that tumors in both models undergo *N-my*c amplification, suggestive of a common pathway to transformation [68].

### "*p107 *single" mice

While *Rb *and *p130 *deletion leads to an increase in Calbindin-positive horizontal cells [68], the horizontal compartment is even further increased in another model in which *Chx10Cre *drives *Rb*^*lox*/*lox *^deletion in *p130-/-, p107+/- *animals, termed "p107 single" [95]. The authors proposed that a differentiated horizontal cell is the retinoblastoma cell-of-origin in this model. An important result from the study is that differentiated horizontal cells with their synaptic processes divide, which was convincingly demonstrated. Interestingly, in *PNMT-Tag *mice, functional pRB family ablation in horizontal cells led to distinct effects depending on retinal topology [54]. *PNMT-Tag *expression led to tumors in peripheral retina but not in central retina, where horizontal cell death eventually occurred; it will be important to carefully examine whether peripheral vs. central horizontal cells also respond differently in *p107-single *mice. While Dyer et al (2007) conclude that the cell of origin is a horizontal cell, the possibility of another cell type that generates the late-stage tumors cannot be ruled out. The metastatic cells in *p107-single *retinoblastomas did not resemble horizontal cells either in ultrastructure or in cell marker expression, suggesting that the retinoblastomas could derive from cells that can produce both differentiated horizontal cells and non-horizontal metastatic cells or that there may be a mixed tumor phenotype with differing cells of origin. This issue is also complicated by the labeling of amacrine cell subsets by horizontal cell markers such as Calbindin and Prox1 [96]. Overall, while the *p107-single *studies clearly show that horizontal cells can divide and raise a very interesting hypothesis regarding a possible cell of origin to the tumors, deletion of these genes specifically in differentiated horizontal cells will be necessary to prove this hypothesis.

### Tumor origins in human and murine retinoblastoma

Differentiated cells are not strong candidates for the cell of origin of human retinoblastoma, given the evidence of multipotent potential to human retinoblastoma cells [32], although one cannot rule out the possibility of dedifferentiation or transdifferentiation occurring. Retinoblastoma occurs over a specific window in time following eye development and is exceedingly rare in adults, suggesting that critical mutational events occur during retinal development. Also, mutation of *RB *is much more likely to occur in a proliferating cell than a post-mitotic cell as DNA replication and cell division can promote an initial gene mutation or loss of heterozygosity. Despite differences in the cell type composition of murine vs. human retinoblastomas, the cell of origin in human and mouse retinoblastoma may both be a similar multipotent cell. That is, the requirement to inactivate *p107 *or *p130 *in addition to *Rb *in the mouse would be expected to mask potential for the murine retinoblastoma cells to exhibit photoreceptor character, as there is widespread death of both rods and cones in the absence of *Rb *and *p107 *or *Rb *and *p130 *when deleted in early progenitors using *Pax6 alpha Cre *[67,68]. Instead, in murine retinoblastoma we observe the characteristics of the cells that best survive *Rb *and *p107 *or *Rb *and *p130 *deletion, i.e. amacrine, Müller and horizontal cells. The specific composition of tumor cell types may be a result of the effects of *p107 *or *p130 *on the survival, proliferation and differentiation of the retinoblastoma cell of origin and its daughters.

### Spatial topology in human retinoblastoma

Observations of early lesions at the extreme retina periphery in mouse retinoblastoma models raised the question of whether spatial differences in the localization of human retinoblastomas occur. Careful examination of retinoblastoma topology has revealed that retinoblastomas that arise in older children tend to occur at the periphery of the retina, while those that occur in younger children are typically central [97,98]. In humans, retinal proliferation ceases in a region of central retina at 14 weeks of gestation and the non-proliferative region expands to the extreme periphery by 30 weeks [26]; this central to peripheral wave of cell cycle exit and differentiation can explain some alterations in retinoblastoma topology with age of detection. Other studies have revealed that new retinoblastomas that arise in patients following treatment for primary retinoblastoma occur much more frequently at the retina periphery. For example, in one study, 15 of 17 new retinoblastomas in bilateral patients were found in the retina periphery [99] (also see [100]. These findings are less easily explained under the common view that retinogenesis ceases near the time of birth. That is, in new retinoblastomas that arise following treatment, the proliferative period of retinal development would have long since ended before the presumptive new tumor arose. Alternative possibilities include the presence of an unappreciated population of progenitors that reside in the retina periphery at a longer time following birth than previously recognized, or a population of peripheral post-mitotic cells that may be capable of transformation. Indeed, there is recent evidence of neuroprogenitors that reside at the periphery of the mamalian retina. In a primate model of myopia, experimental eyelid fusion led to the persistent proliferation of neuroprogenitor cells at the retina periphery months after birth [101]. Of note, control animals injected with BrdU also exhibited proliferation at the retina periphery, although at a much lower level than the stressed animals. These studies revealed proliferation in the juvenile primate retina and modulation with a specific stress. In *patched *heterozygous mice, persistent proliferation of cells expressing the progenitor marker *nestin *was found at the extreme retina periphery in adults, suggesting that the hedgehog pathway may be important for the late proliferating cells [102]. Furthermore, cells with properties of stem cells have been isolated from the pigmented epithelial cells of the murine and human ciliary body [103-105]. The ciliary body is a peripheral eye structure adjacent to neural retina, and close to the location where we first observe retinoblastomas in *α-Cre Rb*^*lox*/*lox *^*p130-/- *or *α-Cre Rb*^*lox*/*lox *^*p107-/- *retinas [68]. Potential neuroprogenitor cells in the mammalian retina may exhibit some similarities to the cells of the ciliary marginal zone in fish and amphibians. In these organisms, retinogenesis continues into adulthood and stem cells clearly reside in the ciliary marginal zone at the retina periphery (reviewed in [106]). It will be critical to investigate the characteristics of the peripheral late proliferating cells in murine retinoblastoma models as this will have important implications for understanding the properties of a retinoblastoma cell of origin.

### A model for retinoblastoma development

An examination of the effects of *Rb *family loss in mouse models suggests a model in which inactivation of *RB *allows the proliferative period of retinal development to be extended in the mutant cells. Upon mutation of both *Rb *and *p107 *or *Rb *and *p130*, this phenotype is exacerbated in mice. This model proposes that homozygous inactivation of *RB *must occur during the normal proliferative period of retinal development, and that this event increases the chance of subsequent mutations by creating a proliferative lesion balanced by cell cycle exit and differentiation, or cell death. The loss of both copies of *RB *may increase the rate of subsequent mutations, as was found in embryonic stem (ES) cells, in which loss of *Rb *led to a dramatic increase in the rate of chromosomal alterations that result in loss of a selectable marker [107]. Loss of *Rb *can lead to chromosome segregation defects through misregulation of the expression of genes important for processes such as centrosome duplication [108,109], mitotic checkpoint control [110] or through impaired maintenance of heterochromatin [111]. In humans, the time between the end of retinal development and the appearance of retinoblastoma may be the time period in which a smoldering lesion caused by homozygous *RB *deletion during development undergoes the secondary mutations needed for tumor formation. In some cases, retinoblastoma may form from a benign precursor lesion, retinoma that exhibits some features of senescence and has recently been shown to exhibit less complex patterns of genetic alteration than adjacent retinoblastoma [112,113]. Interestingly, in retinomas, high levels of p130 were found, which were not observed in adjacent retinoblastoma, suggesting that evasion of p130 might be important for progression of human retinoma to retinoblastoma [112]. One of the essential current questions is the nature of the block to proliferation/tumor expansion that must be overcome even upon homozygous *RB *inactivation. Specifically, is this a differentiation block, or must cell death or other processes be overcome? Examination of the nature of secondary alterations will be needed to shed light on this question.

### Secondary genetic alterations in retinoblastoma

The two-hit hypothesis suggests that the first few years following birth reflects the time-interval for homozygous *RB *mutation [1]. However, there is evidence that *RB *inactivation is not sufficient for retinoblastoma, as cytogenetic studies have revealed consistent chromosomal abnormalities in addition to *RB *deletion [114]. Early cytogenetic studies have been extended by comparative genomic hybridization (CGH) and array-CGH studies. Pooled data across 6 studies reveal recurrent gain in 6p (54%), 1q (53%), 2p (34%) and loss of 16q (32%), suggesting that these regions may harbor oncogenes and tumor suppressor genes, respectively [115-120], reviewed in [121]. The vast majority of these data are from low-resolution studies; the advent of high-resolution oligonucleotide based array-CGH promises to pinpoint minimal regions and identify new, focal regions of change. In murine metastatic retinoblastomas lacking *Rb *and *p130*, array CGH revealed recurrent whole chromosomal gains at chromosome 1 (with synteny to the minimal region of gain in human retinoblastoma at human 1q31-32) and 12 (with synteny to the minimal region of gain at human 2p24) [68]. Such findings of overlapping regions of frequent chromosomal alterations in both murine and human retinoblastoma are encouraging as they help to validate the *α-Cre Rb/p130 DKO *mouse model. While alterations in regions syntenic to human 6p and 16q were not observed in *α-Cre Rb/p130 DKO *retinoblastomas, larger studies are needed, and it is possible that loss of p130 in the murine model may negate selection for 6p gain or 16q loss. Indeed, *RBL2 (p130) *lies at 16q12 and is a good candidate tumor suppressor in this region in humans [121]. Genes in the minimal region of gain at human 6p22 include *DEK *as well as an interesting candidate, *E2F3*, which conceivably could antagonize p130 upon overexpression. Use of mouse models will certainly help in defining the critical genes in these regions, as mouse/human comparisons have revealed highly concordant genomic alterations in other tumor types and have allowed for the identification and validation critical cancer-contributing genes [122,123]. As the topic of genomic alterations in retinoblastoma has recently been comprehensively reviewed [121], here, I will briefly touch upon on the human 1q31-32 and 2p candidate regions that undergo gain or amplification in both murine and human retinoblastomas.

### Human 2p gain/*N-MYC *amplification

The most well characterized secondary alteration in human retinoblastoma is gain or amplification of a region of 2p that includes the *N-MYC *locus [118,124-126]. It is not formally known that *N-MYC *is the gene in the amplicon selected for increased copy number, as other genes are co-amplified, including the DEAD box gene *DDX1*. However, in *α-Cre Rb*^*lox*/*lox *^*p130-/- *metastatic retinoblastomas (3 of 16 metastases), amplicons containing *N-myc *were also observed, ranging from 450 kb to 3.3 MB, and the only known gene in the minimal region of amplification was *N-myc *[68]. This result illustrates the power of high density array-CGH analyses to resolve regions of alteration to single genes and reveals the similarities between human and mouse retinoblastomas. In both murine and human retinoblastoma, low level gain of *N-myc/N-MYC *is found more frequently that high level amplification. The fact that *N-Myc *amplification is enriched in metastatic *α-Cre Rb*^*lox*/*lox *^*p130-/- *retinoblastoma suggests that this event contributes to formation of more aggressive tumors but how this is mediated is unknown. Genetic inactivation of *N-myc *in the developing retina has revealed a critical role for this gene in progenitor cell proliferation; this is in part mediated by regulation of p27 levels [127]. Examination of interactions between *Rb *and *N-myc *in the developing retina and in retinoblastoma models should help us to understand why these mutations synergize in tumorigenesis and to identify the critical *N-myc *target genes.

#### *MDM4 *and the *p53 *pathway in retinoblastoma

Many cytogenetic studies and CGH experiments have demonstrated recurrent frequent gains in 1q [114,121]. While the regions of change typically include large regions of 1q, smaller regions of gain in some tumors have been used to define minimal common regions. Zielinski et al (2005) noted a minimal region centered at 1q32.1-32.2, that included the genes *MDM4 *and *GAC1*, and they also identified a second common minimal region of gain at 1q22 [120]. *MDM4 *is related to *MDM2 *and both genes cooperate to inhibit p53 function, with *MDM4 *an inhibitor of *p53 *transcriptional activity [128]. Laurie et al [59] showed by FISH that *MDM4 *undergoes copy number increase and is overexpressed in retinoblastomas. They report that 65% of human retinoblastomas had extra copies of *MDM4 *and 10% exhibited extra copies of *MDM2*. Furthermore, transfection of *MDM4 *plasmid DNA into newborn retinas *in vivo *led to more rapid tumorigenesis in the *α-Cre Rb/p107 DKO *model. p53 is a good candidate effector downstream of *MDM4 *as homozygous *p53 *loss accelerated retinoblastoma development *in vivo *in *Chx10-Cre; Rb*^*lox*/*lox *^*p53-/-; p107-/- *animals compared to animals with wild-type *p53 *[70,129]. In contrast to these findings, in animals with inactivation of *Rb *on a *p53-/- *background, (*NesCre1 Rb lox/lox p53-/- *mice) retinoblastomas did not result [69], suggesting that *p53 *loss can impact upon retinoblastomas that have already been initiated, but that inactivation of a second pocket protein in addition to *Rb *is still critical. Based on experiments in culture using retinal explants, the authors suggest that suppression of *p53*-dependent apoptosis is crucial [59]. However, murine retinal explants lacking *Rb *exhibit very low levels of apoptosis [73] compared to the high levels observed in *Rb-/- *retinas *in vivo *(Table 2) and therefore may not be an ideal correlate of *Rb*-dependent apoptosis *in vivo*. Indeed, *Rb/p53 *compound mutation did not lead to suppression of cell death associated with *Rb *inactivation *in vivo *[69]. Also, in chimeras with cells lacking *Rb *and *p107*, expression of a dominant negative p53 in *Rb/p107 DKO *photoreceptors using an IRBP-p53DD transgene did not rescue photoreceptor apoptosis *in vivo *[65]. It is also possible that *p53 *loss may contribute to retinoblastoma through deregulation of *p53 *effectors that control the cell cycle or maintain genomic stability. An absence of direct *p53 *mutations in human retinoblastoma suggests that other effectors downstream of *MDM2/MDM4 *may be important. Candidates include the other p53 family members p63 and p73, as well as targets such as Numb and p21 [130-132].

While *MDM4 *is an excellent candidate retinoblastoma oncogene gene on 1q, an important question is whether there are other genes on chromosome 1q that drive selection for 1q gain. Findings of recurrent focal regions of high-level gene amplification at 1q21 and 1q22 [118,133,120] and the identification of additional candidates in the 1q32 minimal region of gain such as the mitotic kinesin *Kif14 *[134,135] suggest that 1q harbors multiple oncogenes. Unlike gliomas, which exhibit high-level focal amplifications of *MDM4 *[136] examination of a large panel of retinoblastomas with 1q gain revealed low-level *MDM4 *gain in retinoblastoma [133]. Low-level *MDM4 *gain, even of a single copy may allow a critical threshold of *MDM4 *signaling to be reached and be physiologically important for tumor development, but other 1q candidates should also be investigated. High-resolution array-CGH experiments using human retinoblastoma samples will help to delineate the regions of focal high-level gene amplifications on 1q most likely to harbor critical oncogenes.

The new data on possible *p53 *pathway involvement in retinoblastoma provides interesting areas for future research and the potential for novel, targeted therapy (reviewed in [137]). Importantly, the addition of Nutlin, an *MDM4/MDM2 *antagonist led to death of retinoblastoma cell lines and synergized with traditional chemotherapy in an orthotopic retinoblastoma mouse model [58,59]. It is clear that radiotherapy can synergize with constitutional *RB *mutation in increasing secondary tumors in patients [12]. The long term effects of systemic chemotherapy on the incidence of secondary tumors in germline *RB *mutant patients are not yet known, but it is conceivable that chemotherapy could contribute to the development of mutations that cooperate with *RB *mutation at sites other than the eye. Also, systemic chemotherapy can lead to debilitating side effects. Thus, localized therapy based on the genetics of known frequent alterations in retinoblastoma combined with localized chemotherapy may provide a safer, effective treatment. It is therefore essential that this pathway be better understood.

## Summary

Research into retinoblastoma has revealed a great deal about a malignant eye cancer, and has also led to many general insights into the general mechanisms of tumor suppression. Early genomic analyses of murine retinoblastoma lacking *Rb *and *p130 *have revealed secondary alterations similar to human retinoblastoma, suggesting that the steps to tumorigenesis in the murine and human retina are similar. Mouse models will be particularly powerful for testing which candidate genes are important for retinoblastoma progression. The novel models provide valuable tools to help us understand the *in vivo *function of pRB, to elucidate the properties of a cancer cell of origin and to test preclinical therapies. We are now poised to gain important insights into the steps between *RB *inactivation in a developing retinal cell and the progression towards retinoblastoma.
